# Supplementary material for: The Vaccinia Virus DNA Helicase Structure from Combined Single-Particle Cryo-Electron Microscopy and AlphaFold2 Prediction
Source: Viruses. 2022 Oct 7;14(10):2206. doi: 10.3390/v14102206 (PMC9611036; doi:10.3390/v14102206)
Supplement: Supplementary file 1 [file viruses-14-02206-s001.zip › viruses-1888170-supplementary.pdf]

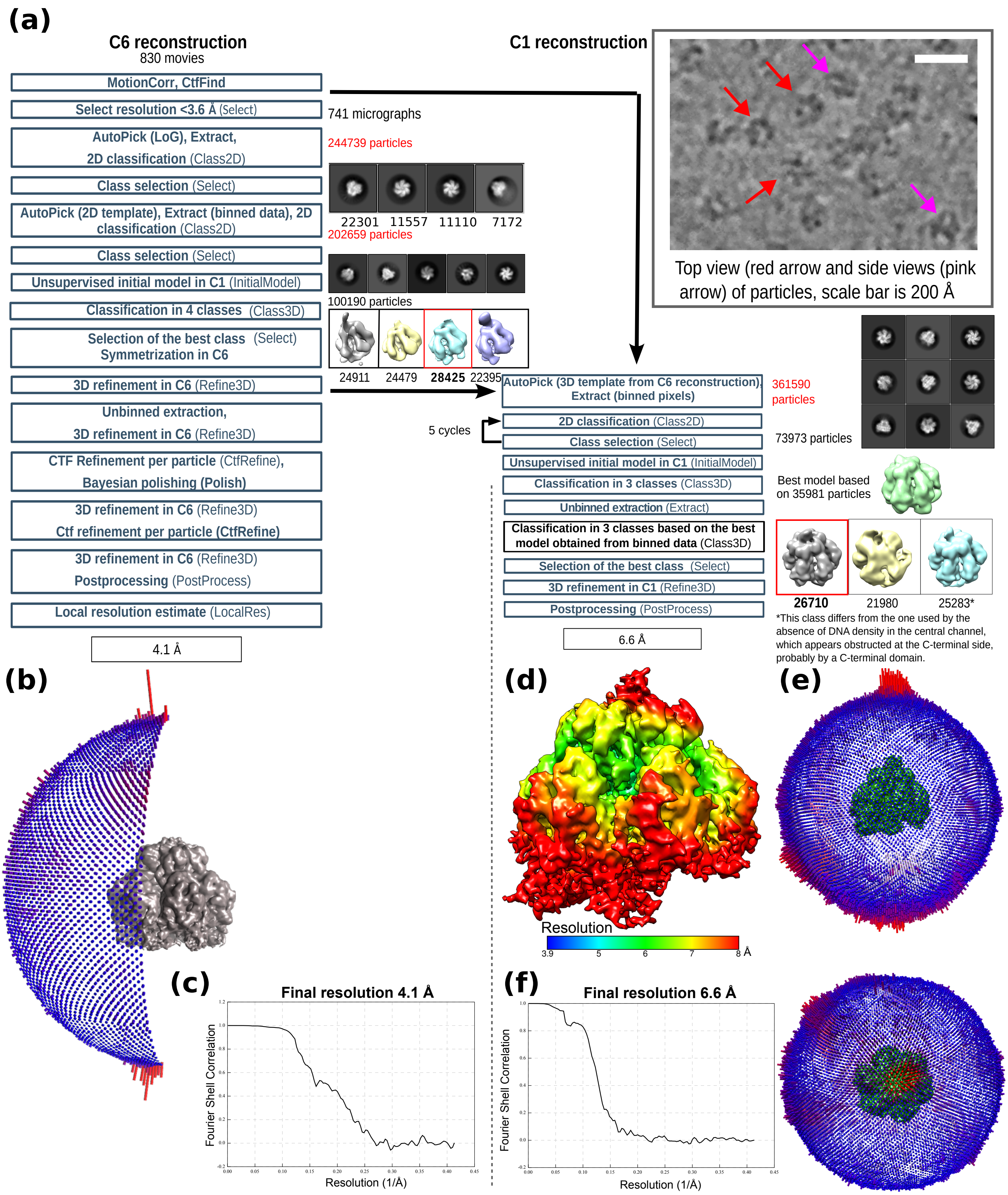

Figure S1. Cryo-EM data processing with Relion 3.1. (**a**) Schematic flow chart of cryo-EM data processing with for particle reconstruction using C6 symmetry (left) and without symmetry (right). Top views of D5$_{323-785}$ particles (red arrows) and side views (pink arrows) in a micrograph (2 µm defocus) are shown on the top right insert. (**b**) Local resolution of the C6 reconstruction. (**c**) Histogram of the orientations of the projections used for 3D reconstruction mapped onto an asymmetric unit in C6 (**d**) Histogram of the orientations of the projections used for 3D reconstruction in C1. (**e**) Fourier shell correlation (FSC) of the reconstruction in C6. (**f**) FSC for the reconstruction in C1.

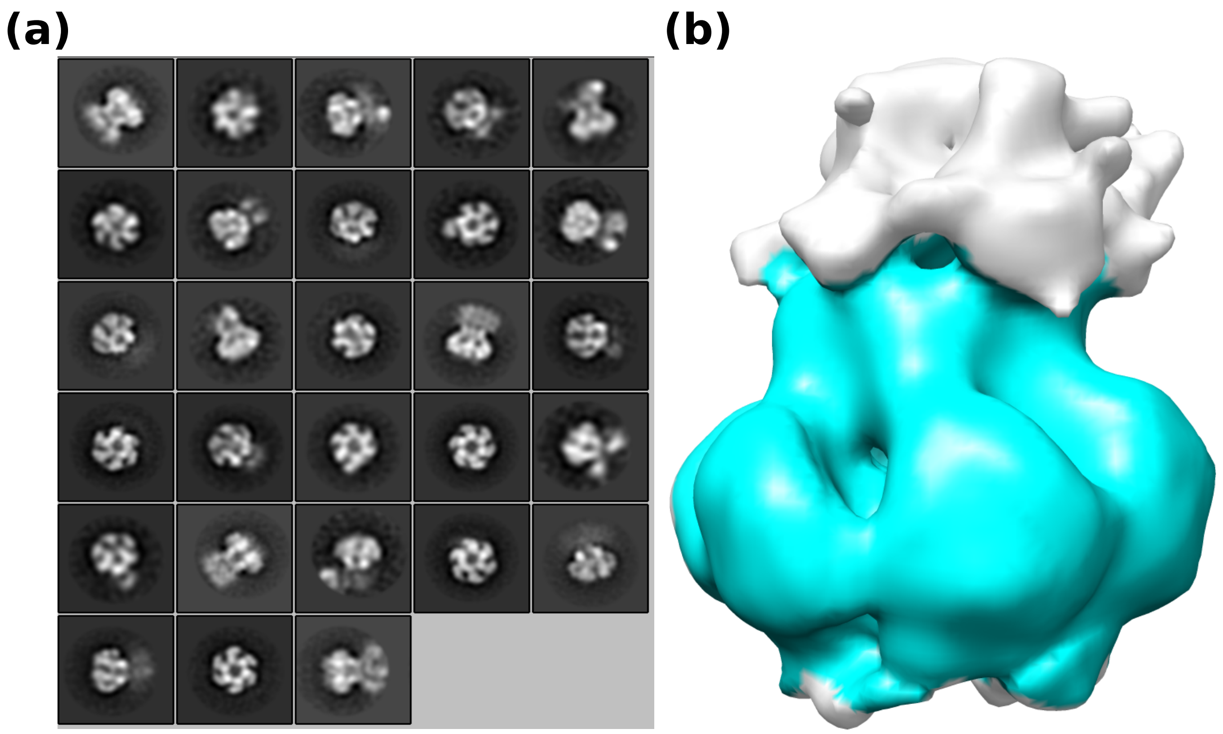

Figure S2. EM of D5fl. (**a**) 2D classes containing 23471 particles of full-length D5 in negative stain. (**b**) Reconstruction of D5fl at 17 Å resolution without imposed symmetry. Density corresponding to reconstructions of $D5_{323–785}$ in negative stain (see Hutin *et al.*, 2016) is colored in cyan. The white density corresponds to disordered primase and Zn-domains.

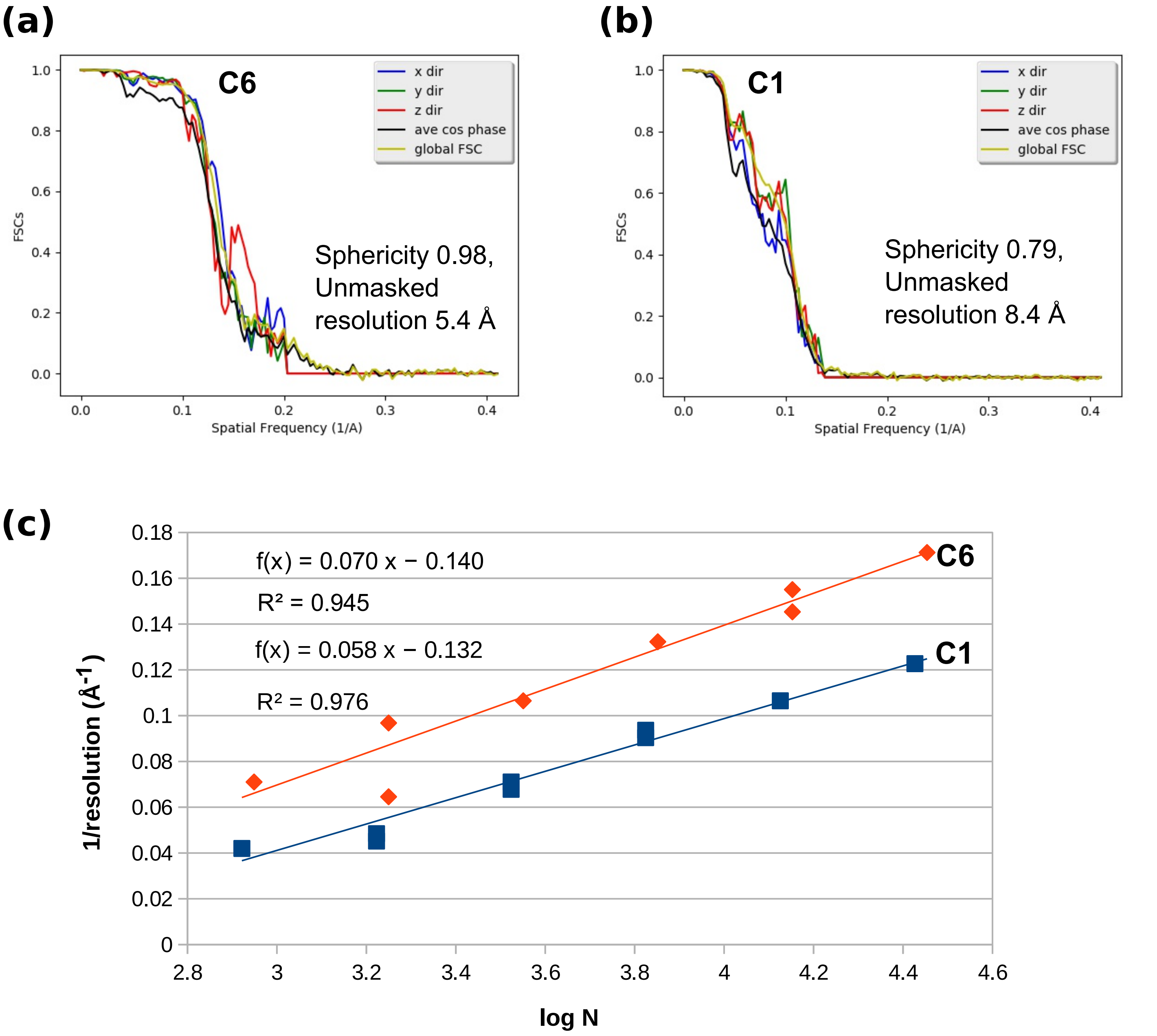

Figure S3. Validation of the final reconstructions. (**a**) Analyses with 3DFSC of the 6-fold symmetric reconstruction and (**b**) the reconstruction without symmetry. **(c)** ResLog analysis of the unmasked reconstruction according to the procedure described by Stagg and co-workers. The particles used for the final reconstruction have been split randomly into 2, 4, 8, 16 and 32 sets. For each number N of particles 2 structures has been refined using Refine3D in Relion. The inverse of the obtained unmasked resolution has been plotted against the log_{10} of the particle number.
